# Evaluating the clinical evidence of TCM in Alzheimer’s disease: an evidence map perspective

**DOI:** 10.3389/fneur.2025.1571361

**Published:** 2025-08-29

**Authors:** Shuqi Cui, Yongli Zhao, Xiaowen Wang, Yingzi Huang, Jiaxi Ye, Ziyong Deng, Yanjiang Li, Hui Qin, Li Wang, Yan Li, Kaihua Wang, Guangshan Zheng, Qijing Qin

**Affiliations:** ^1^Graduate School, Guangxi University of Chinese Medicine, Nanning, Guangxi, China; ^2^International Zhuang Medicine Hospital Affiliated to Guangxi University of Chinese Medicine, Nanning, Guangxi, China; ^3^Guangxi University of Chinese Medicine, Nanning, Guangxi, China

**Keywords:** traditional Chinese medicine, Alzheimer’s disease, randomized controlled trials, meta-analysis, evidence map

## Abstract

**Objective:**

This systematic review aimed to synthesize current clinical evidence from randomized controlled trial (RCT) and meta-analyses on the efficacy and safety of TCM in the treatment of Alzheimer’s Disease (AD).

**Methods:**

Systematic searches across eight biomedical databases (PubMed, Embase, Web of Science, Cochrane Library, CNKI, Wanfang, VIP, SinoMed) through October 26, 2024 yielded an evidence matrix, which was analyzed through integrated narrative-graphic synthesis.

**Results:**

Our analysis encompassed 187 studies (141 RCTs and 46 systematic reviews/meta-analyses), demonstrating cyclical publication growth with recent contraction. Study characteristics included sample sizes of 50–100 participants and intervention durations of 4–24 weeks. Interventions included acupuncture, herbal decoctions, and proprietary medicines. Outcomes focused on clinical efficacy, scale scores, TCM syndrome scores, and safety. While TCM demonstrated therapeutic potential, prescription heterogeneity and diagnostic ambiguity constrained specificity. Methodological quality was generally low, with few high-quality systematic reviews or meta-analyses.

**Conclusion:**

While TCM shows therapeutic potential in Alzheimer’s disease, methodological limitations persist. Subsequent research requires enhanced trial designs with standardized outcome metrics and rigorous bias control protocols.

## Highlights

- This review elucidates the current evidence base for the treatment of Alzheimer’s disease (AD) with Traditional Chinese Medicine (TCM).
- The clinical studies included generally feature relatively small sample sizes.
- A majority of these studies inadequately reflect the principles of syndrome differentiation and treatment, which are fundamental to TCM.
- While TCM appears to hold potential for the treatment of AD, the overall quality of research in this area necessitates substantial improvement.

## 1 Introduction

Alzheimer’s disease, a progressive neurodegenerative pathology, manifests as multidomain cognitive deterioration with core manifestations in mnestic deficits, linguistic processing dysfunction, and executive capacity decline (1, 2). Among neurodegenerative dementias, AD represents a substantial proportion (3). Worldwide, AD prevalence is estimated at 55 million cases, projected to reach 139 million by 2050 (4). An estimated 69 million and 315 million individuals have preclinical and prodromal AD, respectively (5, 6). This large patient population severely burdens socioeconomic systems, family structures, and mental health, establishing AD as a critical global public health challenge (7). Despite limited diagnostic and therapeutic options for AD, RCTs employing TCM interventions demonstrate promising clinical utility with enhanced safety. However, comprehensive evidence validating these methods remains inadequate (8, 9). The evidence map, an emerging methodology, provides a systematic approach to evaluate TCM’s current evidence base, therapeutic potential, and limitations in AD diagnosis and treatment (10). Evidence mapping systematically collates and synthesizes domain-specific literature, enabling comprehensive analysis of research progress and knowledge gaps. This approach provides an empirical foundation for guiding future high-quality studies, significantly advancing TCM applications in AD diagnosis and treatment.

## 2 Materials and methods

### 2.1 Search strategy

A systematic search was conducted across eight databases, including CNKI, WanFang Data, VIP, SinoMed, PubMed, Embase, Web of Science, and Cochrane Library, for literature related to the treatment of dementia with TCM. The search period ranged from the inception of the databases to October 26, 2024. Chinese Search Terms: (“dementia” OR “Alzheimer’s Disease” OR “Alzheimer” OR “AD”) AND (“Traditional Chinese Medicine” OR “Chinese herbal medicine” OR “acupuncture” OR “needle puncture” OR “integrated Chinese and Western medicine” OR “granules” OR “powders”OR “pills” OR “tablets” OR “decoctions” OR “injections” OR “capsules” OR “preparations” OR “oral liquids” OR “injections” OR “tuina” OR “prepared Chinese medicine” OR “external therapy” OR “syndrome typing”) AND (“randomized controlled trials” OR “RCT” OR “systematic analysis” OR “meta”). English Search Terms: (“dementia” OR “Alzheimer” OR “Alzheimer’s Disease” OR “AD”) AND (“Chinese medicine” OR “acupuncture” OR “Integrative Medicine” OR “granules” OR “powder” OR “pill” OR “tablet” OR “decoction” OR “injection” OR “capsule” OR “massage” OR “syndrome”) AND (“randomized controlled trials” OR “RCT” OR “meta-analysis” OR “systematic reviews”).

### 2.2 Inclusion criteria

Eligible studies required a confirmed Alzheimer’s disease diagnosis and were limited to RCTs or meta-analyses/systematic reviews incorporating RCTs. Interventions encompassed Traditional Chinese Medicine (TCM) modalities, including oral/topical herbal compounds (all formulations), proprietary medicines, external therapies (e.g., acupuncture, cupping, moxibustion), or integrative Chinese-Western approaches. Comparator groups involved Western pharmacotherapy, placebo, or other relevant controls, with no restrictions imposed on participant demographics (age, gender), disease duration, comorbidities, or adjunct therapies.

### 2.3 Exclusion criteria

Exclusion criteria comprised preclinical models (animal/cell studies), non-empirical research outputs (case reports, reviews, conference abstracts, expert opinions, patents, technological achievements, media articles), studies investigating vascular dementia or irrelevant pathologies, inaccessible/incomplete datasets, non-anglophone/sinophone publications, and duplicate reports.

### 2.4 Literature screening and data extraction

Five investigators independently executed literature screening and data extraction against predefined inclusion criteria, with cross-validated screening outcomes ensuring methodological fidelity. EndNote X9 facilitated automated deduplication. Title/abstract screening excluded non-conforming studies, followed by full-text appraisal refining the corpus through exclusion of non-compliant or duplicate publications. A data extraction form was established, including but not limited to title, authors, publication date, journal of publication, therapeutic parameters (syndrome stratification strategies, sample size, pharmacotherapeutic protocols, duration), and outcome metrics (TCM syndrome scoring, validated psychometric scales, safety surveillance).

### 2.5 Quality assessment

The methodological quality of the RCTs was assessed using the Cochrane risk of bias tool (11). This tool includes seven domains: (1) random sequence generation; (2) allocation concealment; (3) blinding of participants and personnel; (4) blinding of outcome assessment; (5) incomplete outcome data (attrition bias); (6) selective reporting; (7) other potential sources of bias. Each domain was evaluated as “low risk,” “unclear risk,” or “high risk” based on the information provided in the included studies.

The methodological quality of meta-analyses/systematic reviews was assessed using the AMSTAR 2 tool (12). This tool contains 16 items, with items 2, 4, 7, 9, 11, 13, and 15 being considered key. Each study was rated as “Yes,” “Partially Yes,” or “No” based on the content provided.

The quality assessments were performed by five researchers, and any discrepancies in evaluations were resolved through group discussion, reaching a consensus before finalizing the assessment.

### 2.6 Statistical analysis

The evidence was synthesized through multimodal visualization: Flowcharts delineated literature screening; temporal publication patterns were mapped via chronographic line graphs; bubble matrices quantified intervention-outcome evidence distribution; and network topographies elucidated TCM-acupuncture therapeutic synergies. Methodological rigor was stratified using proportional bar stacking. Quantitative visualizations (line/stacked graphs, bubble matrices) were constructed in Origin 2024, while therapeutic networks were modeled in Cytoscape. Statistical analyses for herbal and acupuncture modalities were computed using SPSS.

## 3 Result

### 3.1 Literature screening process and results

**Figure 1:**
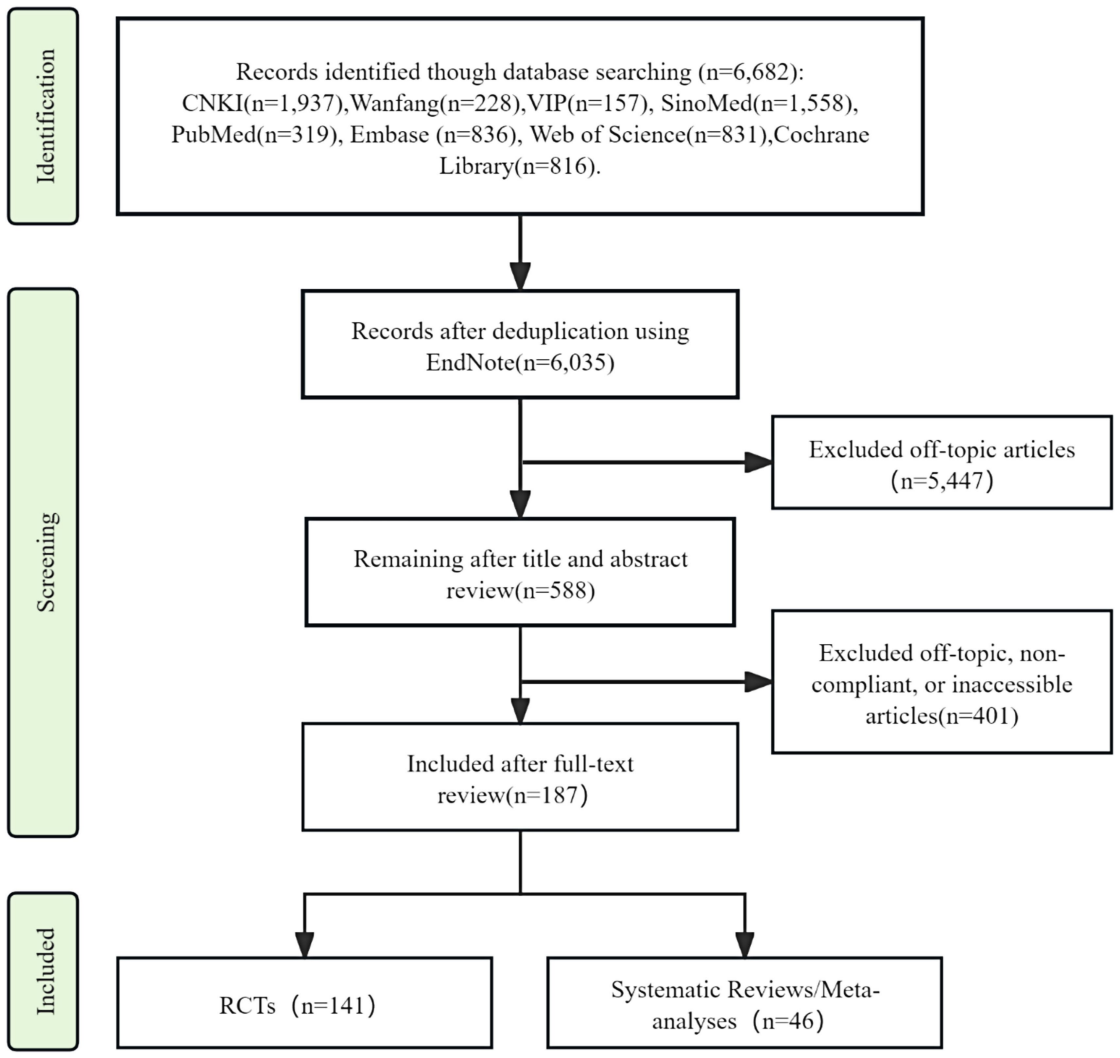
Flow diagram of the literature screening process.

The systematic search yielded 6,682 potentially relevant records. EndNote-assisted deduplication retained 6,035 entries for preliminary evaluation. Title/abstract screening preserved 588 potentially eligible entries. Full-text appraisal confirmed eligibility for 187 studies (141 RCTs; 46 systematic reviews/meta-analyses), with the complete selection protocol detailed in Figure 1.

### 3.2 Trends in publication

**Figure 2:**
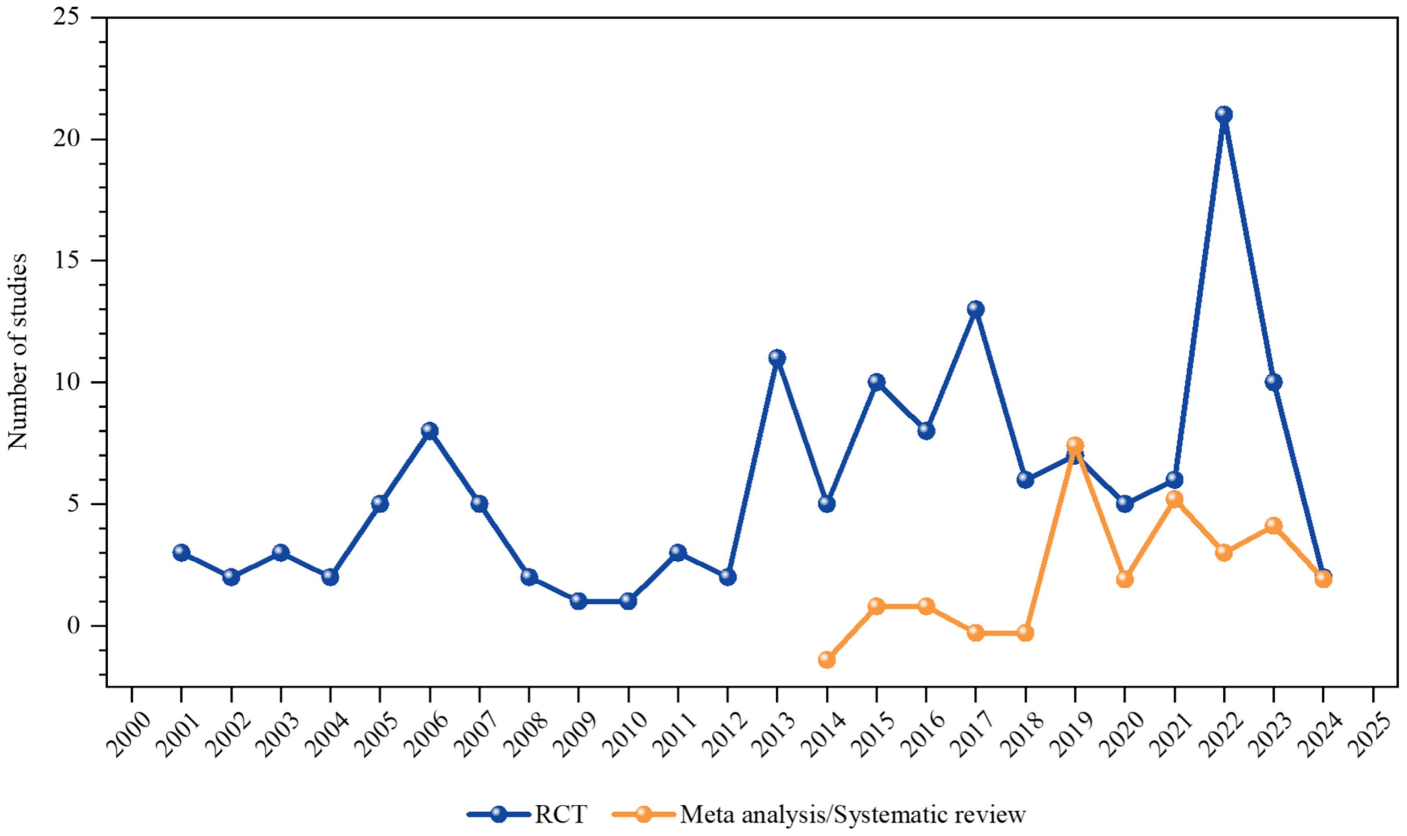
Publication trend graph. The line graph illustrates the number of publications and the trend of publication output from the year 2000 (inclusive) to 2024.

As of October 26, 2024, 141 RCTs and 46 systematic reviews/meta-analyses investigating TCM for AD treatment have been cumulatively published. Publication frequency peaked in 2022 (26 articles), with the lowest output in 2002 (2 articles). The annual publication count has demonstrated a fluctuating but overall upward trajectory since 2000, marked by a notable decline in 2023, as depicted in Figure 2.

### 3.3 Study scale

#### 3.3.1 Sample size

The analysis incorporated 141 RCTs comprising 12,411 participants, with enrollment ranging from 22 to 300 per study. Distribution analysis demonstrated predominant representation (60%) in the 51–100 participant stratum, versus minimal frequency (1%) for cohorts of 200–250 participants. as detailed in Table 1.

**Table 1:** Sample size.

Sample size (*n*)^a^/per	Pub. Vol^b^./per	Fraction^c^/%
*n*<50	21	15%
50<*n* ≤ 100	84	60%
100<*n* ≤ 150	24	17%
150<*n* ≤ 200	6	4%
200<*n* ≤ 250	2	1%
*n*>250	4	3%

a Sample size per RCT.

b A total of 141 RCT publications were categorized by predefined sample size intervals. Collectively, these studies comprised an aggregated participant cohort of 12,411 individuals.

c Proportional distribution of RCT publications stratified by sample size intervals, expressed as a percentage of the total publication cohort.

#### 3.3.2 Source of literature

Among the RCTs included in this study, 28 were master’s theses, 5 were doctoral theses, 5 were published in Trials, 4 in New Chinese Medicine, 4 in Chinese Acupuncture, and 4 in Chinese Medicine Modern Distance Education. Other journals included Liaoning Journal of Traditional Chinese Medicine (3 articles), Sichuan Journal of Traditional Chinese Medicine (3 articles), Chinese and Western Medicine Combined in Cardiovascular Diseases (3 articles), Chinese Journal of Gerontology (3 articles), Chinese Journal of Integrative Medicine on Cardio-Cerebrovascular Disease (3 articles), Journal of Chengdu Medical College (2 articles), Henan Traditional Chinese Medicine (2 articles), Shanxi Journal of Traditional Chinese Medicine (2 articles), World Journal of Traditional Chinese Medicine (2 articles), Chinese Journal of Basic Medicine in Traditional Chinese Medicine (2 articles), Chinese Archives of Traditional Chinese Medicine (2 articles), Journal of Integrative Medicine (2 articles), Guiding Journal of Traditional Chinese Medicine and Pharmacy (2 articles), The institutions such as “Integrative Medicine Research,” “Journal of Acupuncture and Tuina Science,” and “Alzheimer’s & Dementia” only accept one submission each. These sources are presented in Table 2.

**Table 2:** Source of literature.

Source of literature	Pub. Vol./per	Fraction
Master’s theses	28	19.9%
Doctoral theses	5	3.5%
Trials	5	3.5%
New Chinese Medicine,	4	2.8%
Chinese Acupuncture	4	2.8%
Chinese Medicine Modern Distance Education.	4	2.8%
Liaoning Journal of Traditional Chinese Medicine	3	2.1%
Sichuan Journal of Traditional Chinese Medicine	3	2.1%
Chinese and Western Medicine Combined in Cardiovascular Diseases	3	2.1%
Chinese Journal of Gerontology	3	2.1%
Chinese Journal of Integrative Medicine on Cardio-Cerebrovascular Disease	3	2.1%
Journal of Chengdu Medical College	2	1.4%
Henan Traditional Chinese Medicine	2	1.4%
Shanxi Journal of Traditional Chinese Medicine	2	1.4%
World Journal of Traditional Chinese Medicine	2	1.4%
Chinese Journal of Basic Medicine in Traditional Chinese Medicine	2	1.4%
Chinese Archives of Traditional Chinese Medicine	2	1.4%
Journal of Integrative Medicine	2	1.4%
Guiding Journal of Traditional Chinese Medicine and Pharmacy	2	1.4%
Others (1 article)	60	42.5%

### 3.4 Intervention measures

**Figure 3:**
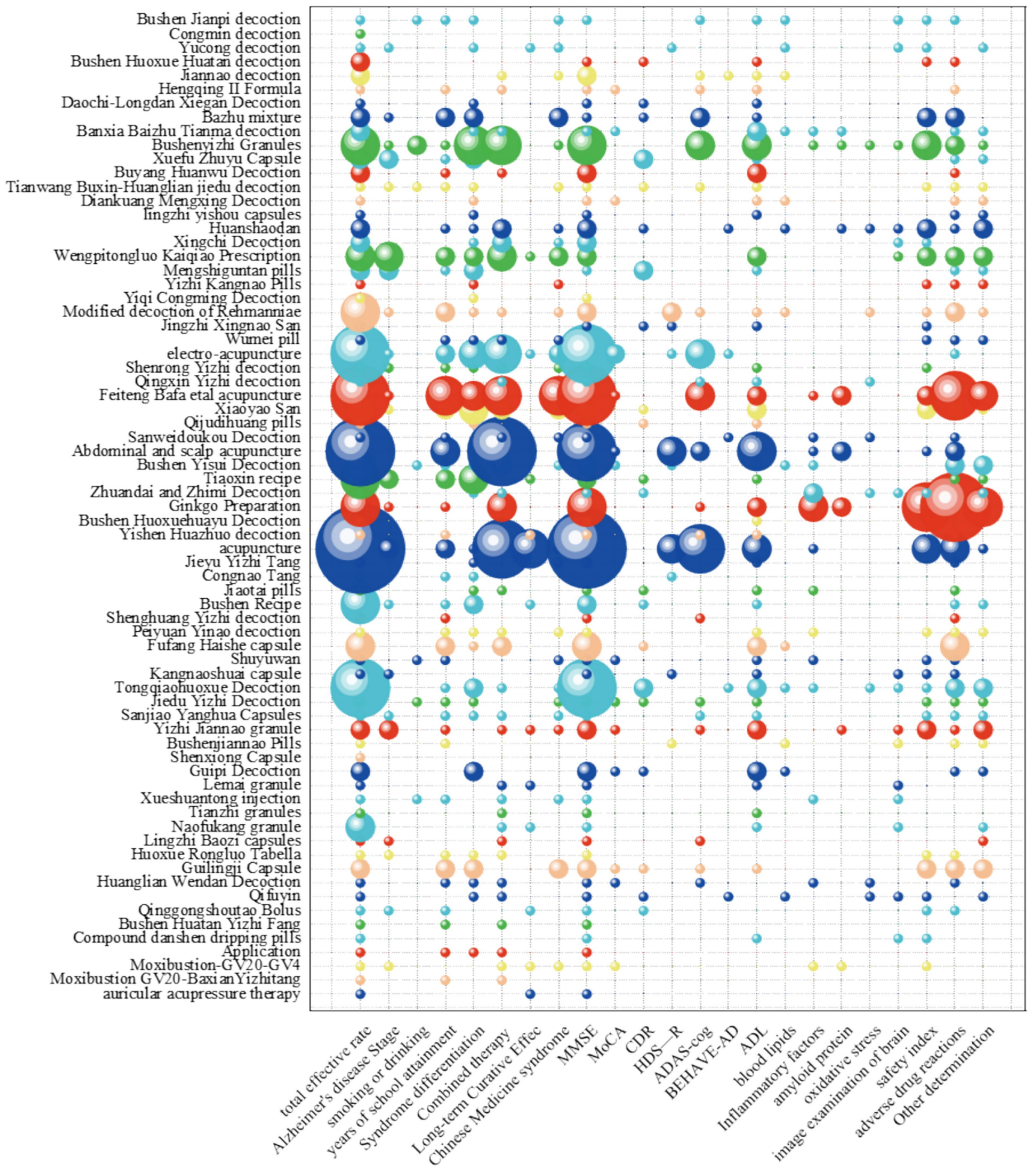
Depicts a bubble chart analyzing the clinical efficacy of traditional Chinese medicine (TCM) in Alzheimer’s disease management. Each bubble’s diameter is proportional to the number of published studies, with color gradients employed to optimize data differentiation and visual interpretability. The x-axis categorizes outcome measures (e.g., cognitive function scores), while the y-axis classifies intervention modalities (e.g., herbal formulae or acupuncture). Notably, TCM interventions predominantly involve complex herbal prescriptions. In clinical practice, physicians adapt these base formulae through herb addition or subtraction (*jiajian*), a therapeutic customization aligned with TCM’s personalized pharmacodynamic principles.

A total of 71 different intervention measures were identified. The most common intervention was the use of Chinese herbal decoctions (61 studies, 43.3%), primarily focusing on kidney-tonifying formulas (17 studies), such as *Bu Shen Huo Xue Fang* (Kidney-tonifying and Blood-activating formula), *Bu Shen Yi Jing Fang* (Kidney-tonifying and Essence-nourishing formula), and *Bu Shen Yi Sui Fang* (Kidney-tonifying and Marrow-nourishing formula), as well as *Tong Qiao Huo Xue Tang* (Blood-activating and Opening the Orifices Decoction) (5 studies) and *Di Huang Yin Zi* (Rehmannia Decoction) (4 studies), among more than 60 other herbal decoctions. Acupuncture combined with other TCM therapies (41 studies, 29%) was also common, along with proprietary Chinese medicines (32 studies, 22.7%), including *Gui Ling Ji Capsule* (3 studies), *Fang Huo Hai She Capsule* (3 studies), and 28 other types. Other interventions (7 studies, 5%), such as poultices, acupressure, auricular acupuncture, and massage, were often used as part of combined therapy, with some not specifically listed. These interventions are summarized in Figure 3.

### 3.5 Influencing factors

A statistical analysis of the sample characteristics provided the following details: Sample age (129 studies)ranged from 45 to 91 years; Disease duration (86 studies) varied from 4 months to 1 year; The stages of Alzheimer’s Disease were clearly defined (28 studies), classified as Stage 1 (mild), Stage 2 (moderate), and Stage 3 (severe); Education (139 studies); Intervention duration (140 studies) ranged from 4 weeks to 1 year; History of smoking and alcohol consumption (10 studies); Syndrome differentiation and treatment (61 studies); Follow-up for efficacy assessment (23 studies).

### 3.6 Outcome measures

Outcome measures across 141 RCTs were stratified by reporting frequency: (1) Total effective rate; (2) Mini-Mental State Examination (MMSE) score; (3) Adverse reactions: dizziness, nausea, vomiting, loss of appetite, sleep disturbances, gastrointestinal symptoms, chest tightness, and bradykinesia; (4) Activities of Daily Living (ADL) score; (5) Safety indicators: complete blood count, urinalysis, stool routine, occult blood in stool, coagulation profile, liver function, kidney function, electrocardiogram, etc.; (6) Other disease-related assessments: laboratory tests, TCM symptoms and signs, etc.; (7) TCM syndrome score; (8) Alzheimer’s Disease Assessment Scale-Cognitive Subscale (ADAS-cog) score; (9) Inflammatory markers: interleukin-1β (IL-1β), interleukin-6 (IL-6), plasma Aβ1-42, tumor necrosis factor-*α* (TNF-α), C-reactive protein (CRP), transforming growth factor-β1 (TGF-β1), etc.; (10) Clinical Dementia Rating (CDR) score; (11) Hasegawa Dementia Scale-Revised (HDS-R) score; (12) Montreal Cognitive Assessment (MoCA) score; (13) Neuroimaging; (14) Amyloid protein levels; (15) Oxidative stress markers; (16) Blood lipids; and (17) Behavioral and Psychological Symptoms of Dementia (BEHAVE-AD) score.

The most commonly reported outcome measures in RCTs for TCM treatment of AD were total effective rate (104 studies) and MMSE score (86 studies), followed by adverse reactions (51 studies) and ADL score (43 studies). Safety indicators (43 studies) and other disease-related measurements (31 studies) were less frequently reported, as summarized in Figure 3.

### 3.7 Frequency analysis of medicinal herb usage

In the included studies, the frequency of individual herbal medicine usage was recorded a total of 2,346 times. The most frequently used herbs in the compound prescriptions were analyzed. The top 15 most frequently used single herbs were: Shichangpu (Acorus tatarinowii Schott) (48 times), Shudi (Rehmanniae Radix Praeparata) (40 times), Yuanzhi (Polygala tenuifolia Willd) (36 times), chuanxiong (Ligusticum chuanxiong Hort) (34 times), Fuling (Poria cocos Wolf) (32 times), Danggui (Angelica sinensis) (29 times), Huangqi (Astragalus membranaceus Bge) (28 times), Shanzhuyu (*Cornus officinalis* Sieb. et Zucc) (27 times), Gancao (Glycyrrhiza uralensis Fisch) (25 times), Yinyanghuo (Epimedium brevicornu Maxim) (25 times), Danshen (*Salvia miltiorrhiza* Bge) (22 times), Heshouwu (*Polygonum multiflorum* Thunb) (22 times), Roucongrong (*Cistanche deserticola* Y.C.Ma) (21 times), Baizhu (Atractylodes macrocephala Koidz) (17 times), and Gouqi (*Lycium chinense* Mill) (17 times).

**Figure 4:**
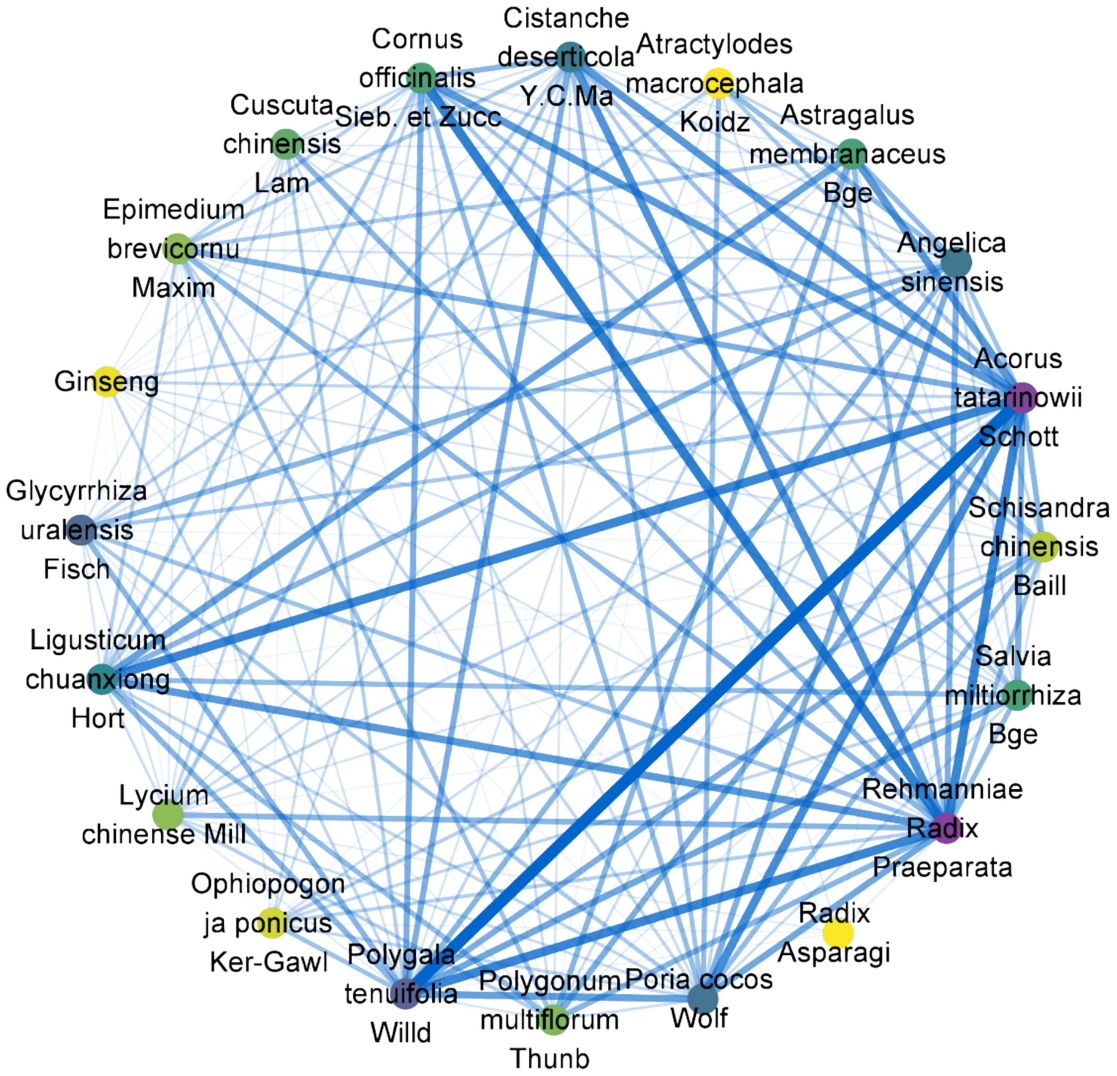
Chinese medicine association rules diagram. Node chromatic intensity denotes TCM utilization frequency, with edge weight reflecting herbal pair co-occurrence frequency.

The top 15 most commonly used herb pairings, based on percentage support, were as follows: Rehmanniae Radix Praeparata—*Cistanche deserticola* Y.C.Ma (20.59%); Acorus tatarinowii Schott—Poria cocos Wolf, Polygala tenuifolia Willd (14.71%); Rehmanniae Radix Praeparata—Bajitian (Morinda officinalis How) (13.73%); Rehmanniae Radix Praeparata—Tusizi (Cuscuta chinensis Lam) (13.73%); Chishao (Paeonia veitchii Lynch)—Taoren (*Prunus persica*(L.)Batsch) (12.75%); Rehmanniae Radix Praeparata—*Cistanche deserticola* Y.C.Ma, Polygala tenuifolia Willd (12.75%); Acorus tatarinowii Schott—*Cistanche deserticola* Y.C.Ma, Polygala tenuifolia Willd (12.75%); Rehmanniae Radix Praeparata—*Cornus officinalis* Sieb. et Zucc, Polygala tenuifolia Willd (12.75%); Acorus tatarinowii Schott—*Cornus officinalis* Sieb. et Zucc, Polygala tenuifolia Willd (12.75%); *Prunus persica*(L.)Batsch—Paeonia veitchii Lynch (11.76%); Glycyrrhiza uralensis Fisch—Chaihu (Bupleurum chinense DC) (11.76%); Acorus tatarinowii Schott—*Cistanche deserticola* Y.C.Ma, *Cornus officinalis* Sieb. et Zucc (11.76%); Polygala tenuifolia Willd—Maidong (Ophiopogon ja ponicus Ker-Gawl) (11.76%); Polygala tenuifolia Willd—Morinda officinalis How, Rehmanniae Radix Praeparata (11.76%); Polygala tenuifolia Willd—Wuweizi (Schisandra chinensis Baill), Acorus tatarinowii Schott (11.76%). These results are summarized in Table 3 and Figure 4.

**Table 3:** Statistical correlations of traditional Chinese medicine herb co-occurrences.

Consequent	Antecedent	Support	Confidence percentage	Gain
Rehmanniae Radix Praeparata	*Cistanche deserticola* Y.C.Ma	20.59	80.95	2.06
Acorus tatarinowii Schott	Poria cocos Wolf, Polygala tenuifolia Willd	14.71	80	1.7
Rehmanniae Radix Praeparata	Morinda officinalis How	13.73	85.71	2.19
Rehmanniae Radix Praeparata	Cuscuta chinensis Lam	13.73	85.71	2.19
Paeonia veitchii Lynch	*Prunus persica*(L.)Batsch	12.75	92.31	7.85
Rehmanniae Radix Praeparata	*Cistanche deserticola* Y.C.Ma, Polygala tenuifolia Willd	12.75	84.62	2.16
Acorus tatarinowii Schott	*Cistanche deserticola* Y.C.Ma, Polygala tenuifolia Willd	12.75	84.62	1.8
Rehmanniae Radix Praeparata	*Cornus officinalis* Sieb. et Zucc, Polygala tenuifolia Willd	12.75	84.62	2.16
Acorus tatarinowii Schott	*Cornus officinalis* Sieb. et Zucc, Polygala tenuifolia Willd	12.75	84.62	1.8
*Prunus persica*(L.)Batsch	Paeonia veitchii Lynch	11.76	100	7.85
Glycyrrhiza uralensis Fisch	Bupleurum chinense DC	11.76	19.67	3.74
Acorus tatarinowii Schott	*Cistanche deserticola* Y.C.Ma, *Cornus officinalis* Sieb. et Zucc	11.76	19.67	1.95
Polygala tenuifolia Willd	Ophiopogon ja ponicus Ker-Gawl	11.76	83.33	2.36
Polygala tenuifolia Willd	Morinda officinalis How, Rehmanniae Radix Praeparata	11.76	83.33	2.36
Polygala tenuifolia Willd	Schisandra chinensis Baill, Acorus tatarinowii Schott	11.76	83.33	2.36

### 3.8 Acupuncture point selection analysis

Analysis of included studies identified 50 acupuncture points employed in AD treatment, with 15 most utilized: Baihui (GV20) (23 times), Sishencong (EX-HN1) (18 times), Shenting (GV24) (15 times), Neiguan (PC6) (11 times), Shenmen (HT7) (11 times), Zusanli (ST36) (11 times), Taixi (KD3) (10 times), Xuan Zhong (GB39) (9 times), Fengchi (GB20) (8 times), Sanyinjiao (SP6) (8 times), Shenshu (BL23) (7 times), Yintang (EX-HN3) (6 times), Benshen (GB13) (5 times), and Dazhui (GV14), Hegu (LI4), Taichong (LR3), Xuehai (SP10), Zhaohai (KI6), and Zhongwan (RN12) (4 times each).

**Figure 5:**
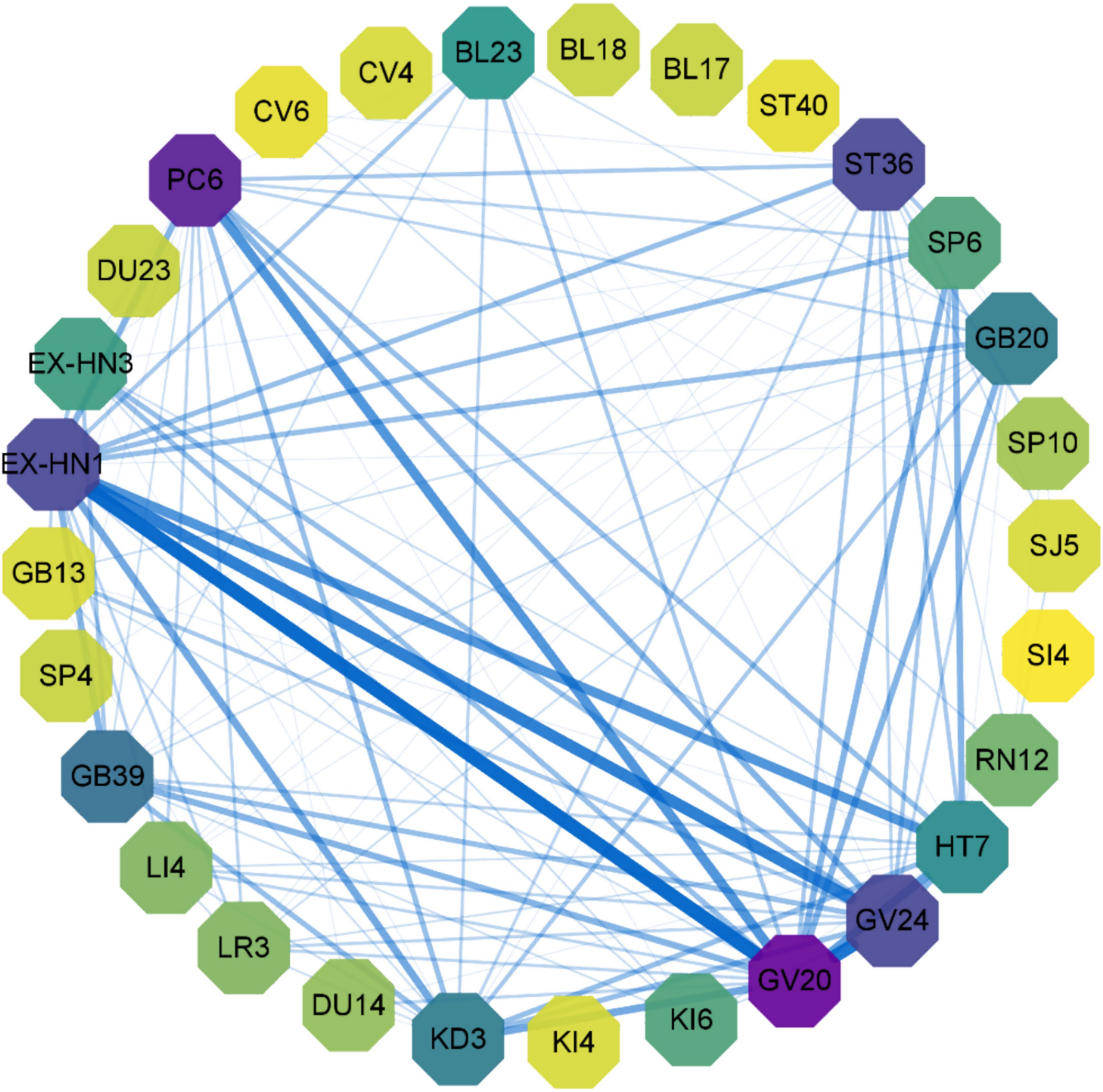
Acupoint association rules graph. Node chromatic intensity denotes acupoint utilization frequency, with edge weight reflecting acupoint co-occurrence frequency.

The top 15 acupuncture point combinations, based on the percentage of support, were: GV20 – GV24 (45.45%); EX-HN1 – GV24 (45.45%); EX-HN1 – GV24, GV20 (39.39%); GV20 – GV24, EX-HN1 (36.36%); EX-HN1 – HT7 (33.33%); GV20 – PC6 (33.33%); GV20 – HT7 (33.33%); EX-HN1 – KD3 (30.30%); GV20 – KD3 (30.30%); GV20 – HT7, EX-HN1 (30.30%); EX-HN1 – HT7, GV20 (27.27%); HT7 – SP6 (24.24%); GV20 – SP6 (24.24%); GV20 – GB20 (24.24%); GV20 – KD3, EX-HN (24.24%); EX-HN1 – KD3, GV20 (24.24%); GV24 – HT7, EX-HN1, GV20 (24.24%). These results are summarized in Table 4 and Figure 5.

**Table 4:** The graphical representation encapsulates the statistical patterns underlying the co-occurrence of traditional Chinese medicine herbs.

Consequent	Antecedent	Support	Confidence percentage	Gain
GV20	GV24	45.45	86.67	1.24
EX-HN1	GV24	45.45	80.00	1.47
EX-HN1	GV24, GV20	39.39	84.62	1.55
GV20	GV24, EX-HN1	36.36	91.67	1.32
EX-HN1	HT7	33.33	90.91	1.67
GV20	PC6	33.33	90.91	1.30
GV20	HT7	33.33	81.82	1.17
EX-HN1	KD3	30.30	80.00	1.47
GV20	KD3	30.30	80.00	1.15
GV20	HT7, EX-HN1	30.30	80.00	1.15
EX-HN1	HT7, GV20	27.27	88.89	1.63
HT7	SP6	24.24	87.50	2.63
GV20	SP6	24.24	87.50	1.26
GV20	GB20	24.24	87.50	1.26
GV20	KD3, EX-HN1	24.24	87.50	1.26
EX-HN1	KD3, GV20	24.24	87.50	1.60
GV24	HT7, EX-HN1, GV20	24.24	87.50	1.93

### 3.9 Methodological quality assessment

#### 3.9.1 Methodological quality assessment of RCTs

**Figure 6:**
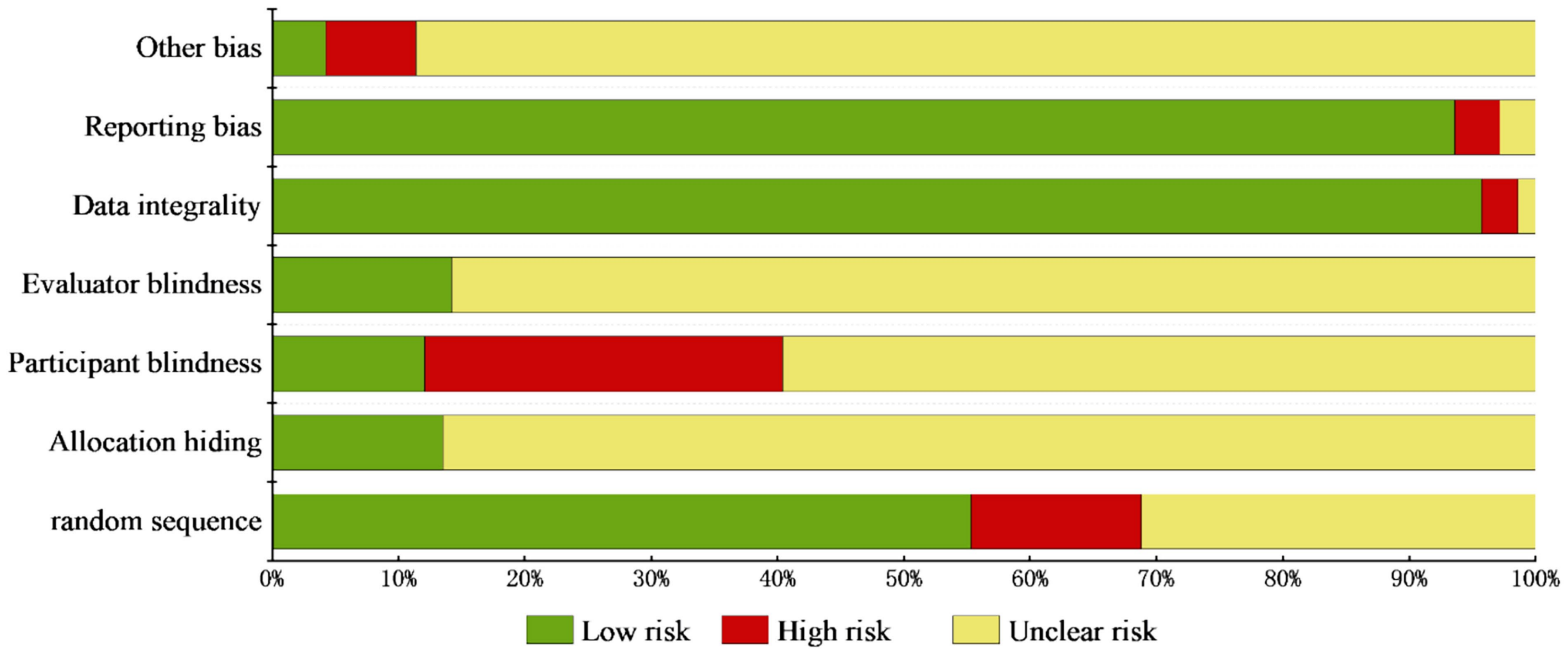
Bar chart representing the percentage of bias risk assessment in included RCTs. The chart utilizes bars of different hues to denote various bias risk domains. The x-axis represents the proportion of studies with different levels of risk, and the y-axis enumerating the seven Cochrane ROB assessment criteria.

Eighteen studies were excluded for incomplete data, yielding 123 studies for final analysis. Risk assessment demonstrated “low bias” in domains including randomization, allocation concealment, participant/personnel blinding, outcome assessment blinding, data completeness, reporting transparency, and other confounders. “Unclear risk” applied to studies with unreported implementation details, while “high risk” indicated absent methodological safeguards, as detailed in Figure 6.

#### 3.9.2 Methodological quality assessment of meta-analyses/systematic reviews

**Figure 7:**
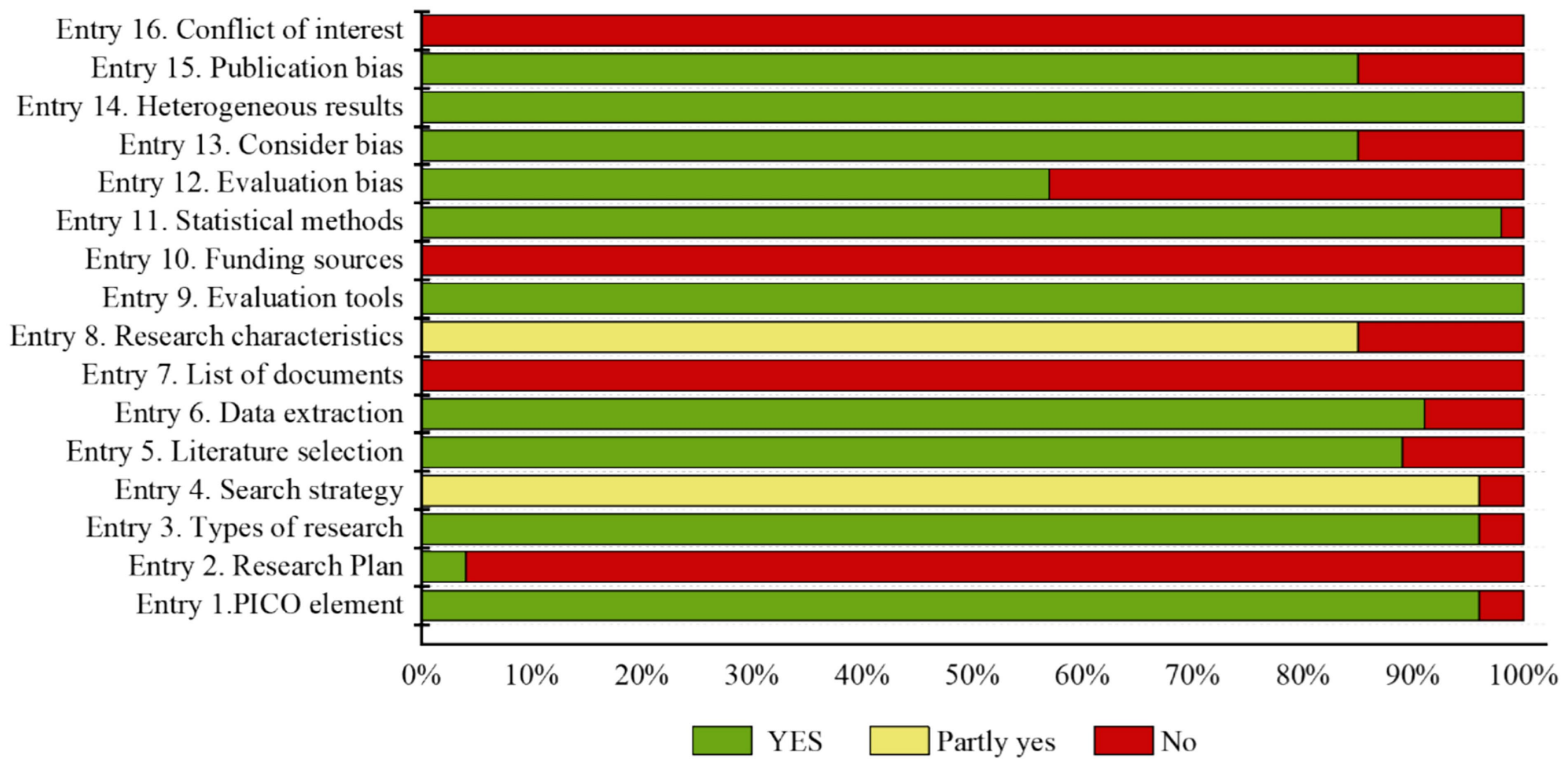
Bar graph illustrating the percentage-based bias risk assessment of included meta-analyses/systematic reviews. The visualization employs chromatic bars encoding distinct bias risk domains, with the x-axis represents the proportion of studies with different levels of risk, the y-axis enumerating the 16 AMSTAR 2 methodological criteria.

The AMSTAR2 tool was used to assess the methodological quality of 46 included meta-analyses/systematic reviews. The results indicated that all included studies employed appropriate methods to assess the risk of bias in each included study, and they reasonably interpreted and discussed the heterogeneity of the research outcomes. However, none of the studies reported the list of excluded studies, funding sources, or conflicts of interest. These findings are illustrated in Figure 7.

## 4 Discussion

This evidence mapping study synthesizes RCTs and systematic evidence syntheses on TCM interventions for AD, delineating the current evidence landscape.

### 4.1 General characteristics of included studies

Quantitative assessment of RCT sample sizes demonstrated 84 trials (60%) enrolled 50–100 participants, whereas 6 trials (4%) encompassed 200–300 participants. Implementation of sample size estimation formulas remains imperative to establish methodologically robust, cost-effective minimum thresholds ensuring trial validity (13, 14). Intervention durations exhibited significant heterogeneity (4 weeks–1 year), with 95% of trials employing ≤24-week protocols, predominantly 8, 12, and 24-week regimens. Furthermore, the evidence base comprised degree thesis (78%), contrasting with limited institutionally indexed articles (22%). This disparity likely stems from the cost-efficiency and smaller-scale feasibility of degree thesis theses versus institutionally mandated studies requiring multicenter collaborations and methodological rigor thresholds. Delayed institutional review cycles and unmet journal impact criteria may further constrain formal dissemination of lower-evidence RCTs (15).

### 4.2 Intervention strategies

The RCTs analyzed in this study indicate therapeutic potential of TCM interventions in AD management. The interventions primarily encompass acupuncture, Chinese herbal decoctions, Chinese patent medicines, moxibustion, and external TCM therapies (e.g., Ear Acupoint Bean Pressing, Herbal Plasters). Acupuncture and herbal decoctions constituted the predominant therapeutic modalities. Among 141 RCTs, 40 (28.37%) adopted acupuncture as the primary intervention, 81(57.44%) adopted traditional Chinese medicine decoctions as the primary intervention, including 11 studies (0.08%) that combined acupuncture with traditional Chinese medicine. Primary acupoints GV20 (Baihui) and EX-HN1 (Sishencong) were selected per Zhenjiu Dacheng (The Great Compendium of Acupuncture) for their bidirectional regulatory capacities in cerebral homeostasis—balancing sedative and nootropic effects. Mechanistic analyses indicate these acupoints modulate cortical neurotransmitter dynamics and neuropeptide homeostasis, enhancing neural circuit plasticity through glutamatergic-GABAergic equilibrium restoration (16, 17).

Herbal decoctions for AD predominantly featured Tongqiao Huoxue Decoction, Dihuang Yinzi, and customized formulations. Given the compositional heterogeneity of bespoke prescriptions, analytical focus centered on high-frequency herbal dyads and monocomponent agents. The most prevalent phytotherapeutic pairs included Rehmanniae Radix Praeparata—*Cistanche deserticola* Y.C.Ma; Acorus tatarinowii Schott—Poria cocos Wolf, Polygala tenuifolia Willd; Rehmanniae Radix Praeparata—Morinda officinalis How. Radix Rehmanniae Preparata is known for nourishing kidney yin (18), *Cistanche deserticola* Y.C.Ma supplements kidney yang and enhances essence and blood (19), Acorus tatarinowii Schott opens the orifices and clears the mind (20), Poria cocos Wolf strengthens the spleen and calms the heart (21), and Polygala tenuifolia Willd improves memory and calms the mind (22). *Morinda officinalis How* demonstrates therapeutic efficacy through dual mechanisms of renal yang tonification and osteoarticular reinforcement (23). The Rehmanniae Radix Praeparata—*Cistanche deserticola* Y.C.Ma dyad, chronicled in Yizong Bidu (Essential Readings of Medical Tradition), achieves cerebrotonic effects through yin-yang equilibrium. Modern mechanistic studies validate its capacity to mitigate senescence-associated cognitive decline via pleiotropic modulation of neuroinflammatory and neurotrophic pathways (24), while Acorus tatarinowii Schott has anti-inflammatory and antioxidant effects (3, 25). Polygala tenuifolia Willd exhibits nootropic efficacy and neuroprotective properties through multi-target pharmacological modulation (26).

### 4.3 Outcome indicators

Outcome measures constitute fundamental determinants of clinical research validity (27). This investigation revealed outcome measurement limitations: Firstly, evidence standardization deficits, with merely 28 studies operationally defining AD staging criteria, and as the disease progresses, patients’ cognitive functions and abilities in daily living gradually decline, which may significantly impact outcome measure (28). Only 10 studies documented lifestyle (e.g., smoking/alcohol use) risk factors implicated in oxidative-inflammatory pathophysiology that accelerates neurodegeneration/apoptosis, thereby influence AD therapeutic efficacy (29, 30). Only 23 articles recorded long-term follow-up for efficacy, leading to a lack of data on long-term efficacy and potential issues; And Evidence deficits persist in family/past medical history. Secondly, Within TCM therapeutic frameworks, only 14 studies operationally defined bianzheng criteria—the diagnostic paradigm governing TCM therapeutic strategies. This methodology enables etiopathogenic precision through syndrome-specific intervention tailoring, ensuring TCM-consistent clinical evidence generation (31). Lastly, There are some limitations in the evaluation indicators of therapeutic effects: Sparse implementation of TCM-specific symptomatology assessments; Non-standardized adverse event documentation via TESS protocols; commonly used scales, such as the MMSE, ADAS-cog, HDS-R, and ADL, Heterogeneity in assessment criteria may introduce variability in therapeutic outcomes across studies (32). Future clinical research should prioritize rigorous trial design validation, metric standardization, and systematic integration of TCM-specific biomarkers including symptomatology quantification and syndromic stratification to enhance methodological robustness and evidentiary credibility.

### 4.4 Methodological quality assessment

Literature quality assessment constitutes a methodological cornerstone in systematic reviews, ensuring methodological rigor for evidence-based clinical trial design and guideline formulation (33). Cochrane bias assessment of included RCTs demonstrated predominant unclear risk ratings, suggesting suboptimal methodological quality requiring standardization enhancement. Methodological deficiencies, including deficient randomization and allocation concealment protocols, compromised participant/investigator blinding integrity, potentially introducing placebo effects. Concurrent investigator preferentiality and assessor expectancy bias may collectively introduce measurement inaccuracies that undermine therapeutic effect validity. In terms of study design and implementation, most RCTs have incomplete information, lacking key data such as sample size calculation, long-term efficacy follow-up, and the number of dropouts. The 46 meta-analyses/systematic reviews demonstrate significant outcomes of TCM in treating AD, but the overall credibility is not high. Critical deficiencies encompass absent prospectively registered protocols, omission of excluded study inventories, insufficient declaration of funding provenance, and opacity in conflict-of-interest disclosures. Strengthening research validity requires implementation of rigorous randomization procedures, adherence to evidence-based frameworks, incorporation of longitudinal outcome tracking, enhancement of evidentiary integrity, and systematic mitigation of bias contamination (34).

### 4.5 Recommendations for future research

Building upon the identified evidence gaps, we recommend four key methodological improvements to advance future studies: (1) stringent implementation of bias-control strategies, including randomization and blinding procedures; (2) utilization of standardized reporting tools, such as TCM syndrome scoring systems; (3) integration of trial designs with the fundamental tenets of TCM syndrome differentiation and treatment; and (4) extension of follow-up durations to evaluate long-term therapeutic outcomes. Collectively, these refinements are designed to strengthen both the methodological rigor and translational relevance of future TCM-based interventions for AD.

## 5 Conclusion

This evidence mapping study evaluated randomized controlled trials and systematic reviews of Traditional Chinese Medicine interventions for Alzheimer’s disease. While demonstrating therapeutic potential, critical methodological limitations were identified. Underpowered sample cohorts and inherent challenges in blinding manual interventions such as acupuncture contributed to clinical heterogeneity. Additionally, the studies failed to report confidence intervals for clinical efficacy and inadequately addressed methodological heterogeneity. The evidence framework remains incomplete, with insufficient representation of TCM’s syndrome differentiation and treatment principles and a lack of distinct TCM-specific outcome measures.

Methodological appraisal revealed prevalent design flaws including ambiguous randomization protocols and suboptimal blinding implementation, undermining validity. Future research priorities should emphasize large-scale multicenter trials adhering to standardized TCM guidelines, development of syndrome-specific therapeutic protocols, and actively exploring and incorporating TCM-specific evaluation criteria and efficacy indicators is essential for a more comprehensive assessment of TCM’s therapeutic efficacy and safety in treating AD. These initiatives seek to establish a rigorous evidence base validating TCM’s therapeutic efficacy in Alzheimer’s disease management.

## Data Availability

The original contributions presented in the study are included in the article/supplementary material, further inquiries can be directed to the corresponding author.
